# Does Closing the Donut Hole Reduce Financial Burdens Among Medicare Part D Beneficiaries?

**DOI:** 10.1093/geroni/igab046.3266

**Published:** 2021-12-17

**Authors:** Yalu Zhang, Lan Liu, Jingjing Sun, Xinhui Zhang, Jiling Sun, Xinming Song, Gong Chen

**Affiliations:** Peking University, Beijing, Beijing, China (People's Republic)

## Abstract

The Medicare Part D donut hole has been gradually closed since 2010. But it is still unclear how it has impacted the beneficiaries’ relative financial burdens, especially in the later stage of the closing plan. The measurement of catastrophic health expenditure induced by prescription drugs (CHE-Rx) reflects the relative financial burdens to beneficiaries’ household income, which bears more information than the measure of dollar-value expenses or the absolute poverty line used in prior studies. Using the Medical Expenditure Panel Survey 2008-2017 longitudinal national representative data and the method of difference-in-differences, this study found that the donut hole closing policy was associated with more usage of prescription drugs (b=2.84, p=0.023) and a higher likelihood of experiencing CHE-Rx (b=2.4%, p=0.011) among those who fell in the donut holes. Besides, the results show that the donut hole closing policy did not generate any immediate effects on prescription drug usage, CHE, and CHE-Rx. For the first time, this paper examined both the aggregated and marginal impact of the policy implementation, which had closed by an additional 35% between 2013 and 2017, on the relative financial burden among the beneficiaries.

